# Genetic and Functional Dissection of the *NFKB2* Gene: Implications for Milk Fatty Acid Biosynthesis in Dairy Cattle

**DOI:** 10.1096/fj.202501411R

**Published:** 2025-07-01

**Authors:** Lijun Shi, Zijiao Guo, Nan Shen, Cong Li, Bo Han, Dongxiao Sun

**Affiliations:** ^1^ Department of Animal Genetics and Breeding, College of Animal Science and Technology, Key Laboratory of Animal Genetics, Breeding and Reproduction of Ministry of Agriculture and Rural Affairs, National Engineering Laboratory for Animal Breeding, State Key Laboratory of Animal Biotech Breeding China Agricultural University Beijing China; ^2^ Institute of Animal Science Chinese Academy of Agricultural Sciences Beijing China

**Keywords:** dairy cattle, EMSA, fatty acids, knocked‐down of *NFKB2*, transcriptional activity

## Abstract

Milk fat, an essential component of bovine milk, significantly impacts human health and dairy industry profitability. Our previous research identified that the *NFKB2* gene was a key candidate for milk fatty acids (FAs) in dairy cattle. This study aimed to investigate the role of *NFKB2* in regulating milk FAs in Holstein cattle through post‐GWAS analysis. Knockdown of *NFKB2* using siRNA in bovine mammary epithelial cells (BMECs) significantly reduced triglyceride concentrations, lipid droplet secretion, and the expressions of 17 key genes involved in lipid metabolism pathways, including PI3K‐AKT, NF‐κB, MAPK, adipocytokine, PPAR, and AMPK signaling pathways. These findings highlighted *NFKB2* as a critical regulator of milk FA composition through its interactions with key lipid biosynthesis pathways. Further, this study constructed a new Chinese Holstein population including 1065 cows and performed genotyping and milk FAs measurements in these individuals. By SNP identification and association analysis, SNP g.22889812C/T in the 5′ flanking region of *NFKB2* was significantly associated with multiple milk FAs, including higher C14:1, C17:0, C18:0, and C14 index levels in allele C group, and higher C6:0, C8:0, C14:0, C16:1, and C16 index levels in allele T group. Using computational predictions, luciferase assays, EMSA and super‐shift EMSA, we revealed that SNP g.22889812C/T downregulated *NFKB2* transcription activity with allele T selectively binding with transcription factor (TF) ZNF282. This study not only advanced the understanding of genetic mechanisms underlying milk fat production but also identified *NFKB2* and its causal mutation g.22889812C/T as promising molecular markers for selective breeding to optimize milk fat traits.

## Graphical abstract

In this study, *NFKB2* was verified to have a significant genetic effect on FAs. Knockdown of *NFKB2* significantly reduced triglyceride concentrations, lipid droplet secretion, and the expressions of 17 key genes involved in lipid metabolism pathways, including PI3K‐AKT, NF‐κB, MAPK, adipocytokine, PPAR, and AMPK signaling pathways. Causal mutation g.22889812C/T in the 5′ flanking region of *NFKB2* downregulated *NFKB2* transcription activity with allele T selectively binding with transcription factor ZNF282.

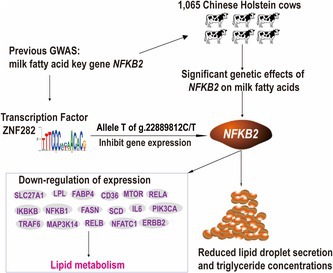

**Abbreviations**
BMECs: bovine mammary epithelial cells
E2F7: E2F transcription factor 7
EMSA: electrophoretic mobility shift assay
FAs: fatty acids
GWAS: genome‐wide association study
HEK: human embryonic kidney
MYT1: myelin transcription factor 1
NFAT5: nuclear factor of activated T‐cells 5
NFKB2: nuclear factor kappa B subunit 2
PRDM4: PR domain zinc finger protein 4
qRT‐PCR: quantitative real‐time PCR
QTLs: quantitative trait loci
SFAs: saturated fatty acids
SNPs: single nucleotide polymorphisms
TF: transcription factor
TFBS: transcription factor binding site
UFAs: unsaturated fatty acids
ZNF282: zinc finger protein 282
ZNF35: human zinc finger protein ZNF35

## 1 Introduction

Milk and milk products, containing numerous essential nutrients, are nutritious food items [1]. Bovine milk is essential for human nutrition; its fat content frequently faces scrutiny as a potential contributor to cardiovascular disease [2]. Milk fat, as one of the most important breeding objectives in dairy cattle, contains a diverse composition of saturated and unsaturated fatty acids (UFAs) [3]. Fatty acids (FAs), including saturated fatty acids (SFAs) and UFAs, present in milk fat are recognized as essential nutritional components in the diets of a substantial segment of the global human population, playing a significant role in influencing human health outcomes [4]. The biological activity of FAs exhibits structural dependency, with carbon chain length, saturation degree, and stereoisomerism determining their different roles in metabolic regulation and cellular signaling pathways [5, 6, 7, 8, 9]. In dairy cattle, the heritability of milk FAs in Holstein cows has been estimated—namely, 0.07–0.42 [10, 11, 12, 13, 14, 15].

Genome‐wide association study (GWAS) is a valuable approach used to uncover possible genetic variations that play a role in significant complex traits in both humans and domesticated animals. For the milk yield and composition traits, lots of candidate genes have been detected by GWAS in dairy cattle [16, 17, 18, 19, 20]. For the milk FAs, several GWAS had detected the functional genes and quantitative trait loci (QTLs), such as *CYP17A1*, *ACO2*, *NT5C2*, *STAT5A*, *GH*, *FASN*, *PCYT2*, *DCXR*, *G6PC3*, *PYCR1*, and chromosome region of BTA15 (22.6–29.0 Mb) [2, 21, 22, 23]. In our initial GWAS for milk FAs in Chinese Holstein cows, a total of 83 genome‐wide significant single nucleotide polymorphisms (SNPs) were identified [24], in which, the SNP ARS‐BFGL‐NGS‐107403 (rs109915272) was strongly associated with C14:1 (*p* = 2.22E‐12) and C14 index (*p* = 2.62E‐15). Further, by scanning for the candidate genes nearby to this significant SNP, *NFKB2* (nuclear factor kappa B subunit 2, ID 526392) gene was identified as the most promising candidate, in which, the SNP rs109915272 was located on the up‐stream regulatory region with distance of 1586 bp. In addition, *NFKB2* gene has been found to be associated with milk protein and fat percentages traits [25].

Nuclear factor kappa B subunit 2 (*NFKB2*) gene encodes a subunit of the transcription factor complex nuclear factor‐kappa‐B that is the endpoint of a series of signal transduction events related to many biological processes such as inflammation, immunity, differentiation, cell growth, tumorigenesis and apoptosis. It was involved in NF‐kappa B, MAPK and breast cancer signaling pathways. Of note, MAPK regulates the growth of mammary cells, and their proliferation and survival [26]. Therefore, this study aimed to further investigate whether the *NFKB2* gene had significant impacts on milk FAs, and elucidate the regulatory mechanisms of *NFKB2* underlying milk FAs, thereby providing valuable genetic markers for genomic selection programs in dairy cattle.

## 2 Materials and Methods

### 2.1 Validation of *NFKB2* Gene Function at Bovine Mammary Epithelial Cells (BMECs)

#### 2.1.1 Transfection of siRNAs in BMECs

Two small interfering RNAs (siRNAs) targeting the *NFKB2* gene (*NFKB2*‐1128 with the sense of 5′‐GGACACCUCCCUAUCACAATT‐3′ and *NFKB2*‐576 with the sense of 5′‐CCCACAGUCUGGUGGGCAATT‐3′) were designed and synthesized in GenePharma Biotech (Suzhou, China). The BMECs were cultured in DMEM‐F12 (DMEM Nutrient Mixture F‐12, Gibco, Life Technologies, United States) with 10% FBS at 5% CO_2_ and 37°C as our previous study [27]. Further, using the X‐tremeGENE siRNA Transfection Reagent (Roche, Penzberg, Germany), we transfected the *NFKB2*‐targeting siRNAs and NC siRNAs into BMECs. After transfection of 48 h, the cells were collected to be measured for phenotypes and frozen in liquid nitrogen for RNA extraction. All biological replicates of the experiments consisted of three samples.

#### 2.1.2 RNA Extraction, Reverse Transcription and Quantitative Real‐Time PCR (qRT‐PCR)

The total RNA of BMECs was isolated with the Trizol reagent (Invitrogen, Carlsbad, CA, USA). The quantity and quality of RNA were measured by NanoDrop 2000 spectrophotometer (Thermo Scientific, Hudson, DE, USA) and gel electrophoresis, respectively. The reverse transcription was conducted by PrimerScriptH RT reagent Kit (TaKaRa Biotechnology, Dalian, China). With Primer 3 version 4.0, 18 pair of quantitative primers for 18 genes (*SLC27A1*, *LPL*, *FABP4*, *CD36*, *MTOR*, *IKBKB*, *NFKB1*, *RELA*, *FASN*, *SCD*, *IL6*, *PIK3CA*, *CDKN1A*, *TRAF6*, *MAP3K14*, *RELB*, *ERBB2*, and *NFATC1*) involved in NF‐kappa B, MAPK, Adipocytokin, PI3K‐Akt, AMPK, and PPAR signaling pathways were designed. Also, the quantitative primers of *NFKB2*, *GAPDH*, and *MAVADL1* were designed. We performed the quantitative real‐time PCR (qRT‐PCR) through SYBR green fluorescence (Roche, Penzberg, Germany) with a volume of 15 μL consisting of 2 μL template of cDNA, 0.375 μL of each primer (10 mM), 4.75 μL distilled water, and 7.5 μL SYBR Green Mixture. The PCR conditions were denaturation 95°C for 10 min; amplification 45 cycles at 95°C for 10 s, 58°C for 10 s, and 72°C for 10 s. The relative expressions of *NFKB2* and its pathway‐related genes were normalized by the *GAPDH* and *MAVADL1* with 2^−ΔΔCt^ method. Each measurement was performed in triplicate.

#### 2.1.3 Phenotypic Measurement in the NFKB2 Knocked‐Down BMECs

This research evaluated the triglyceride concentration before and after *NFKB2* knocked‐down in BMECs using the glycerol lipase oxidase (GPO‐PAP) method with a Triglyceride Assay Kit (Nanjing Jiancheng Biotechnology Institute, Nanjing, China). In this assay, triglycerides underwent a series of chemical reactions to produce red quinone, and the optical absorbance at 510 nm was directly proportional to the triglyceride content. Additionally, the lipid droplet secretion was assessed by the Oil Red O Stain Kit (Nanjing Jiancheng Biotechnology Institute). In this procedure, lipids were stained bright red, nuclei were stained dark blue, and other cellular components were stained light blue.

## 3 Identification and Mechanism Analysis of *NFKB2* Causative Mutation Affecting Milk Fatty Acids

### 3.1 Animals and Milk Fatty Acid Phenotypes

To verify whether *NFKB2* gene indeed affects the milk fat traits in dairy cows, this study constructed a new Chinese Holstein population including 1065 individuals as a validation group. In this study, milk samples of 2 mL were gathered from these Chinese Holstein cows from 44 sire families, which were raised in 23 dairy farms of Beijing Dairy Cattle Center. A total of 16 milk FAs were measure by gas chromatography [24], including C6:0, C8:0, C10:0, C11:0, C12:0, C13:0, C14:0, C14:1, C15:0, C16:0, C17:0, C17:1, C18:0, C18:1cis‐9, and C20:0. In addition, we calculated SFA and UFA as well as SFA/UFA, and derived five indices using the equation

$$\begin{array}{c} \frac{\mathrm{cis}-9\;\text{unsaturated}}{\mathrm{cis}-9\;\text{unsaturated}+\text{saturated}}\times 100\;:\;C14\text{index}=\frac{C14:1}{C14:1+C14:0}\\ \times 100,C16\text{index}=\frac{C16:1}{C16:1+C16:0}\times 100,C17\text{index}\\ =\frac{C17:1}{C17:1+C17:0}\times 100,C18\text{index}=\frac{C18:1\mathrm{cis}-9}{C18:1\mathrm{cis}-9+C18:0}\\ \times 100,\text{and total index}\\ =\frac{C14:1+C16:1+C17:1+C18:1\mathrm{cis}-9}{C14:1+C14:0+C16:1+C16:0+C17:1+C17:0+C18:1\mathrm{cis}-9+C18:0}\\ \times 100. \end{array}$$

Also, this study collected the genomic DNA from semen samples of 44 sires and blood samples of 1065 cows using the TIANamp Blood DNA kit (Tiangen Biotech, Beijing, China). DNA quantity and quality were assessed with a NanoDrop 2000 Spectrophotometer (Thermo Scientific, DE, USA) and 1% gel electrophoresis respectively. The semen‐derived DNA samples were pooled into two equal groups (*n* = 22 per pool), normalizing each to a final concentration of 50 ng/μL before proceeding with downstream analyses.

### 3.2 SNP Identification and Genotyping

To amplify the entire coding region and 3000 bp of up‐/down‐stream flanking sequences, 23 pairs of primers were designed with Primer 3 version 4.0 based on the genomic sequence of the bovine *NFKB2* gene (Gene ID 526392) and performed the synthesis in the Beijing Genomics Institute (Beijing, China). Further, we conducted the PCR amplification using the pooled DNA as the template by the following procedures initial denaturation at 94°C for 5 min, followed by 35 cycles at 94°C for 30s; 60°C for 30s; 72°C for 30s; and a final extension at 72°C for 7 min. The PCR amplification products were bi‐directionally sequenced using an ABI3730XL DNA analyzer (Applied Biosystems, Foster, CA, USA) to identify potential polymorphisms.

Subsequently, 1065 cows were genotyped on each identified SNP by using the matrix‐assisted laser‐desorption/ionization time of flight mass spectrometry (MALDI‐TOF MS, Sequenom MassARRAY, Bioyong Technologies Inc., HK).

### 3.3 Association Analysis

Using SAS 9.2, the phenotype–genotype association analyses between the identified SNP and 24 milk FA traits was conducted with the mixed animal model below,

$$Y_{\text{ijklm}}=\mu +G_{i}+h_{j}+l_{k}+a_{l}+b\times M_{m}+e_{\text{ijklm}}$$

In which, $Y_{\text{ijklm}}$ was the phenotypic value of each milk FA trait; μ was the overall mean; $G_{i}$ was the fixed effect corresponding to the genotype combination of individual *i*; $h_{j}$ (*j* = 1–23) and $l_{k}$ (*k* = 1–4) were the fixed effect of farm *j* and stage of lactation *l*, respectively; $a_{l}$ was the random polygenic effect; $M_{m}$ (*m* = 1–293) was the fixed effect of age at calving *m*; b was the regression coefficient of covariate M; and $e_{\text{ijklm}}$ was the random residual. We created the kinship matrix (A‐matrix) for association analysis by tracking the 1065 cows back to three‐generation pedigrees with the SAS 9.2 (SAS institute, Cary, NC, USA), and 3335 individuals in total were included.

Correspondingly, according to $\begin{array}{c} ɑ=\frac{\mathrm{AA}-\mathrm{BB}}{2},d=\mathrm{AB}-\frac{\mathrm{AA}+\mathrm{BB}}{2},\text{and} \end{array}$ $\begin{array}{c} \alpha =ɑ+d\left(q-p\right) \end{array}$, the additive effect (a), dominant effect (d), and allele substitution effect (*α*) were respectively calculated as well. The genotype‐corresponding MFA least square means among them were AA, AB, and BB, and the frequencies of alleles A and B were p and q, respectively.

### 3.4 Prediction of Transcription Factor (TFs) Changed by the SNP g.22889812C/T

To determine whether the mutation g.22889812C/T affected the binding of transcription factors, Genomatix software suite v3.9 [28] was utilized for transcription factor prediction by the default parameters.

#### 3.4.1 Recombinant Plasmid Construction and Transfection Luciferase Assay

**FIGURE 1:**
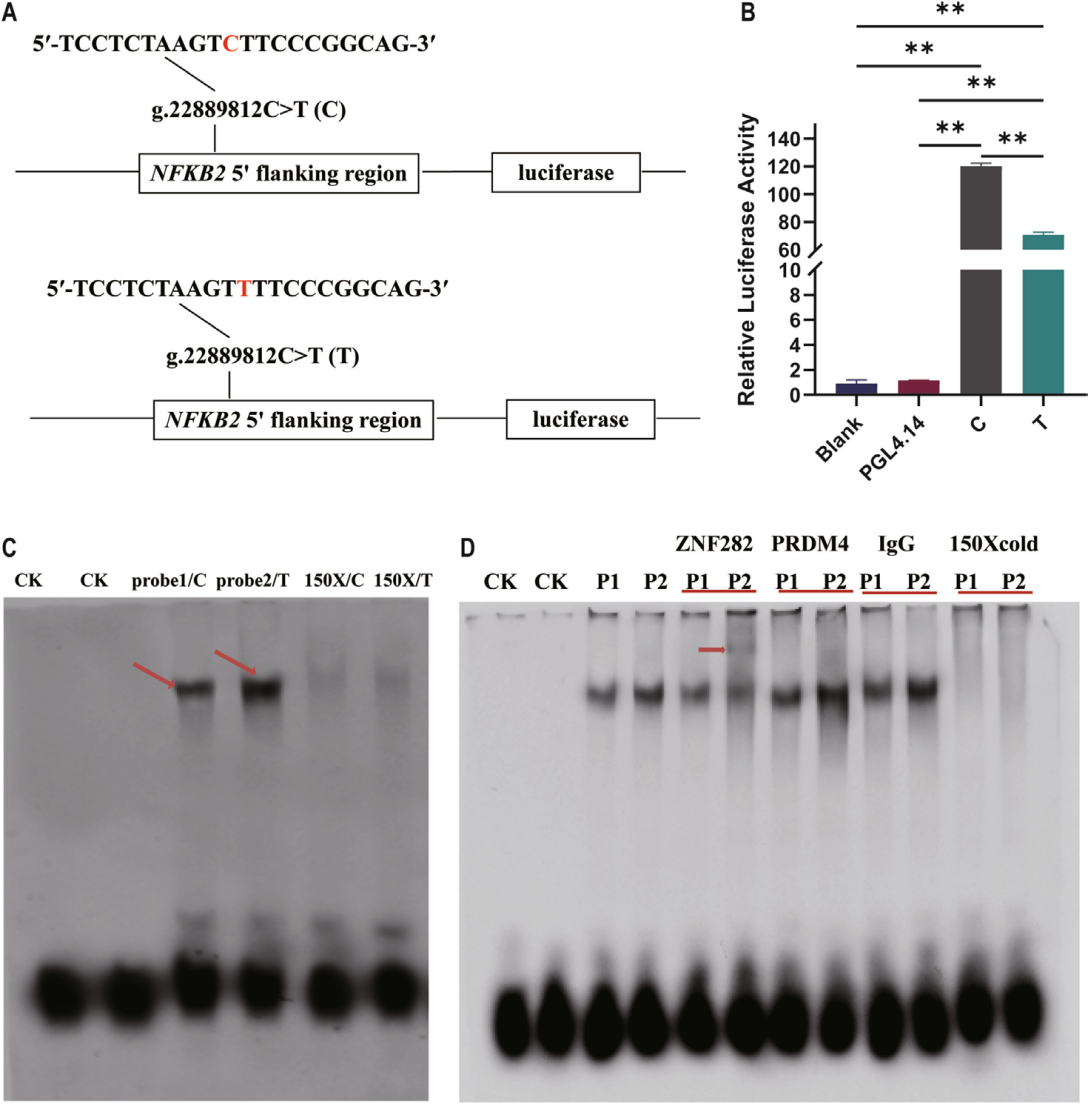
Identification transcription factor (TF). (A) Transient reporter gene expression assays were conducted using constructs featuring the g.22889812C/T (rs109915272) variant in the *NFKB2* gene, with the SNP in the sequences highlighted in red. (B) Luciferase activity analysis was performed on the recombinant plasmids incorporating the g.22889812C/T (rs109915272) variant in HEK‐293T cells. The reported values for each construct represent the mean of three independent experiments, each conducted in triplicate. ** denotes *p* < 0.01. (C) Electrophoretic mobility shift assay (EMSA) was conducted using C and T oligonucleotides corresponding to the g.22889812C/T (rs109915272) site, with CK representing the blank control. The 150‐fold excess of an unlabeled consensus oligonucleotide probe was utilized, indicated as 150×. (D) Super‐shift analysis of g.22889812C/T (rs109915272) was performed to validate the specific TF predicted to be ZNF282. CK represented nuclear extracts without oligonucleotide probes. Lanes 3 and 4 illustrated the DNA‐binding capacities of the alleles in the absence of antibody. Lanes 5 and 6 showed the DNA‐binding abilities of the alleles when bound to the ZNF282 antibody, while lanes 7 and 8 depicted the binding capabilities with PRDM4 antibody. Lanes 9 and 10 demonstrated the DNA‐binding abilities when using IgG antibody. Lanes 11 and 12 corresponded to the use of 150× unlabeled consensus oligonucleotide probes. P1 denoted the probe containing the allele C of g.22889812C/T (rs109915272), and P2 signified the probe with the allele T.

To further verify whether the SNP g.22889812C/T changed the promoter activity of *NFKB2*, we designed two luciferase reporter gene fragments, representing the C and T alleles of g.22889812C/T (Figure 1A), incorporating KpnI and HindIII restriction sites at the 5′ and 3′ termini, respectively. These fragments were cloned into the pGL4.14 luciferase assay vector (Promega, Madison, USA) and synthesized by them (Genewiz, Suzhou, China). Subsequently, this experiment purified all the plasmids using the Endo‐free Plasmid DNA Mini Kit II (OMEGA, omega bio‐tek, Norcross, Georgia, USA) and sequenced the products to confirm the integrity of the insertions in each construct.

Human Embryonic Kidney (HEK)‐293T cells were cultured in DMEM (Dulbecco's modified Eagle's medium; Gibco, Life Technologies, United States) with 10% FBS (fetal bovine serum; Gibco), 100 U/mL penicillin (Gibco) and 100 mg streptomycin (Gibco) at 5% CO_2_ and 37°C. Approximately 2 × 10^5^ HEK 293 T cells were seeded into a 24‐well plate, and the plasmids were HEK 293 T cells. All the experiments were performed in triplicate.

This experiment harvested the cells at 48 h post‐transfection and assessed the activity of both firefly and Renilla luciferases using the Dual‐Luciferase Reporter Assay System (Promega) on a Modulus microplate multimode reader (Turner Biosystems, CA, USA). The normalized luciferase activity data (Firefly/Renilla) was calculated by averaging the results from the three replicates.

#### 3.4.2 Electrophoretic Mobility‐Shift Assay (EMSA) and Super‐Shift Assay

To assess the impact of the SNP g.22889812C/T on DNA‐binding affinity, an Electrophoretic Mobility Shift Assay (EMSA) was conducted using biotinylated oligonucleotide probes containing the SNP (5′‐TTCCTCTAAGTC/TTTCCCGGCAGCCGGGCC‐3′). Nuclear extracts were prepared from the mammary gland cells of lactating Holstein cows using the NE‐PER Nuclear and Cytoplasmic Extraction Reagents (Thermo Scientific), and nuclear protein concentration was quantified using a BCA reagent kit (Pierce, Thermo Scientific) with a Microplate Reader at 562 nm (DX800, Paradigm, Molecular Devices, Silicon Valley, California, USA). The EMSA was performed following the manufacturer's instructions (Pierce), utilizing a 150‐fold excess of non‐radioactive probes as competitors. A native 6% DNA retardation gel was employed to assess changes in mobility, thereby confirming whether the SNP g.22889812C/T altered TF binding.

Through prediction analysis, the SNP g.22889812C/T could alter transcription factor binding. Based on the ci‐value and *p*‐value, two most probable transcription factors, namely, ZNF282 and PRDM4, were identified. Hence, we conducted a super‐shift EMSA using specific antibodies of ZNF282 and PRDM4 to determine which one was affected by the mutation. Additionally, an IgG antibody was used as a control. Like the EMSA, nuclear extracts from mammary cells were utilized for the experiment, employing cold probes at a concentration of 150‐fold excess.

## 4 Results

### 4.1 *NFKB2* Knocked‐Down in BMECs Inhibited the Lipid Secretion

**FIGURE 2:**
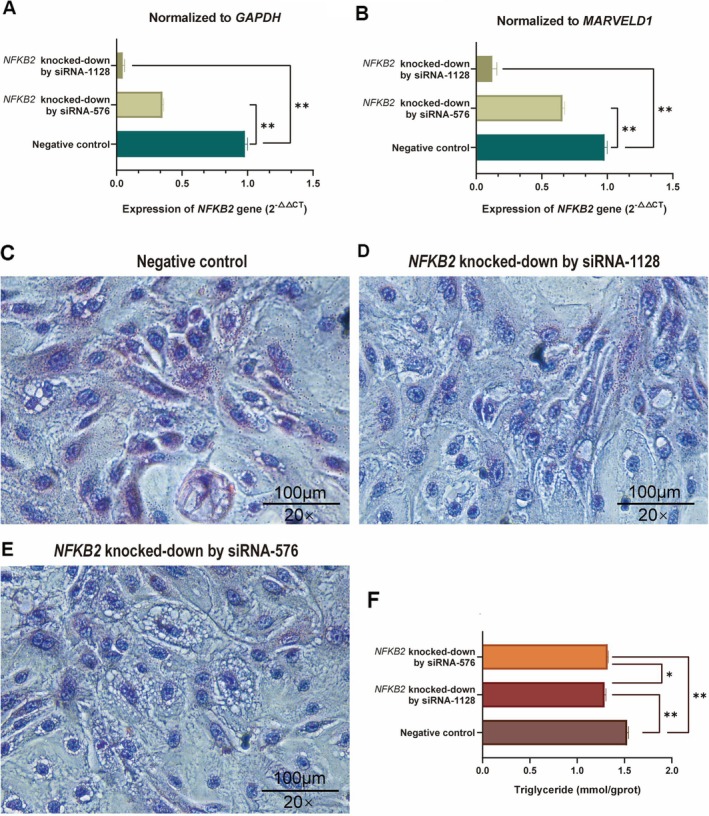
Effect of *NFKB2* knocked‐down on lipid secretion in BMECs. (A) Relative expression levels of *NFKB2* normalized to *GAPDH* before and after knockdown. (B) Relative expression levels of *NFKB2* normalized to *MARVELD1* before and after knockdown. (C) Lipid secretion profile in BMECs prior to *NFKB2* knockdown. (D and E) Lipid secretion profiles in BMECs following *NFKB2* knockdown. (F) Changes in intracellular triglyceride concentrations in BMECs after *NFKB2* knockdown. * and ** denote *p* < 0.05 and *p* < 0.01, respectively.

To investigate the role of *NFKB2* in milk fat metabolism, this research employed interference techniques in BMECs to evaluate its effect on lipid secretion. Following the knockdown of *NFKB2* using siRNA‐576 and siRNA‐1128, a significant reduction in *NFKB2* expression (*p* < 0.001, Figure 2A,B) was observed. The significant reduction in triglyceride concentration within the BMEC cells (*p* ≤ 0.0032, Figure 2F) was shown in the knockdown of *NFKB2* groups. Further, this study analyzed lipid secretion alterations using the Oil Red O Stain Kit and found that *NFKB2*‐knockdown BMECs exhibited a marked decrease in the secretion of lipid droplets compared to both blank and negative control cells (Figure 2C–E). The siRNA‐1128 more effectively reduced *NFKB2* expression (*p* < 0.001, Figure 2A,B) and triglyceride concentration (*p* < 0.05, Figure 2F) than siRNA‐576, and also resulted in a more evident reduction in lipid droplet secretion (Figure 2C–E). These findings indicated the *NFKB2* gene promoted milk lipid synthesis.

**FIGURE 3:**
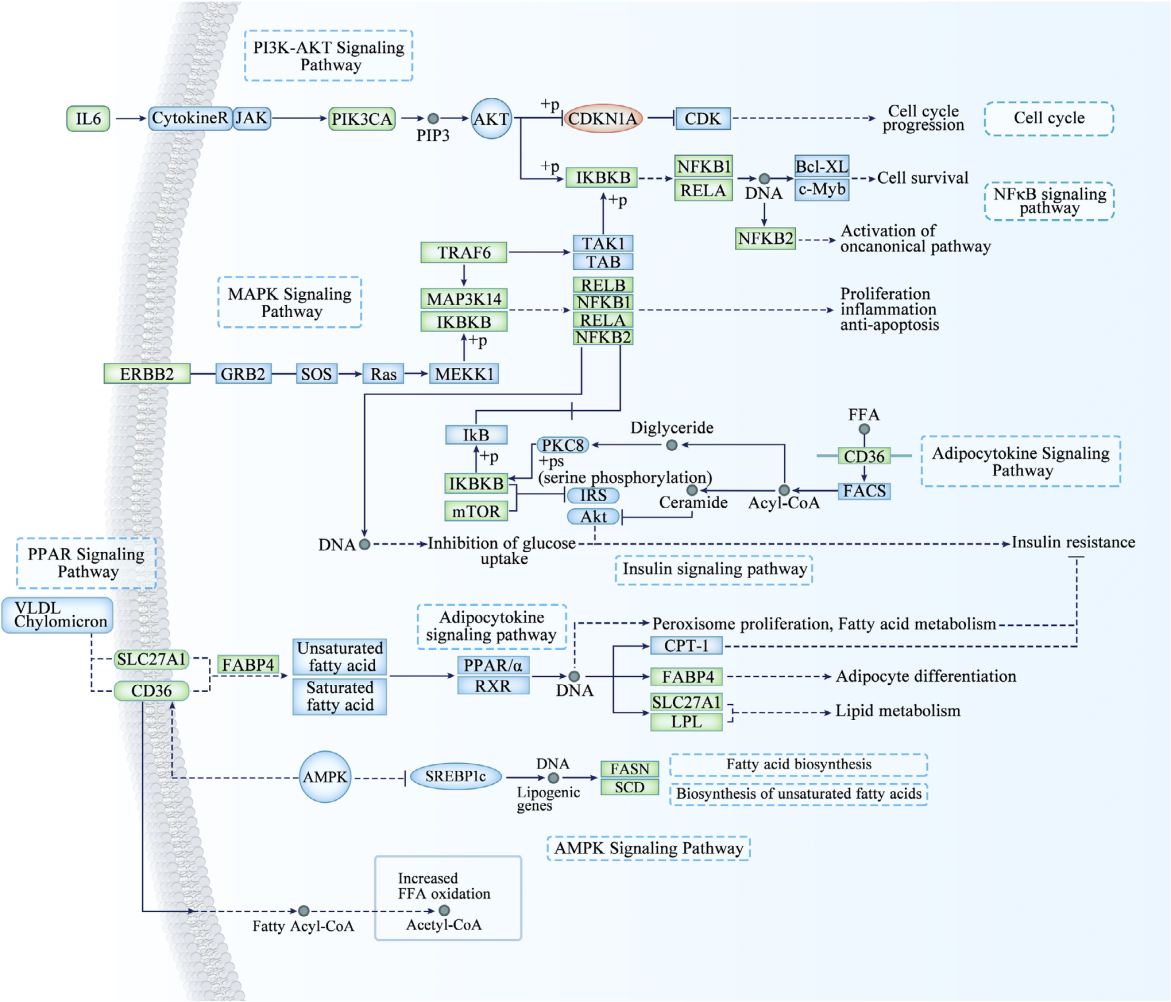
Differentially expressed genes following *NFKB2* knockdown in BMECs were found in PI3K‐AKT, NF‐κB, MAPK, adipocytokine, PPAR, and AMPK signaling pathways. Downregulated genes after *NFKB2* knockdown were indicated by green frames, and the upregulated genes were highlighted in brownish red.

To elucidate the mechanisms by which *NFKB2* influences milk lipid synthesis, we assessed the expression levels of 18 genes that were primarily involved in PI3K‐AKT, NF‐κB, MAPK, adipocytokine, PPAR, and AMPK signaling pathways with qRT‐PCR, including *SLC27A1*, *LPL*, *FABP4*, *CD36*, *MTOR*, *IKBKB*, *NFKB1*, *RELA*, *FASN*, *SCD*, *IL6*, *PIK3CA*, *CDKN1A*, *TRAF6*, *MAP3K14*, *RELB*, *ERBB2*, and *NFATC1* genes. We observed the mRNA levels of these genes were significantly reduced following the knocked‐down *NFKB2* gene compared to the control group except *CDKN1A* (*p* < 0.05, Figure 3), implying the *NFKB2* gene might influence the metabolism of milk FAs through various key lipid pathways. According to the changes of these gene expressions, the main mechanism map (Figure 3) of *NFKB2* gene regulation of milk FAs was constructed.

### 4.2 SNP Identification in *NFKB2* Gene

By screening the entire coding and 3000 bp of up−/down‐stream flanking sequences of *NFKB2* gene, a SNP (g.22889812C/T) was identified in the dairy cattle population, which was located within 5′ flanking region of *NFKB2* gene. Of note, it was found strongly associated with C14:0 and C14 index in our previous GWAS [24]. In this study, the genotypic and allele frequencies for g.22889812C/T were presented in Table 1.

**TABLE 1:** The information of SNP g.22889812C/T (rs109915272) in *NFKB2* gene.

SNP name	Location	Position (UMD3.1.1)	GenBank no.	Genotypes	No.	Frequency	Allele	Frequency
rs109915272	5′ flanking region	Chr2622889812	rs109915272	CC	460	0.4423	C	0.6688
				CT	471	0.4529		
				TT	109	0.1048	T	0.3313

*Note:* No. was the number of animals involved in corresponding genotype.

### 4.3 Associations of SNP g.22889812C/T With 24 Milk Fatty Acids

The association analyses between SNP g.22889812C/T and 24 milk FAs were performed. Our results revealed that g.22889812C/T was significantly associated with C6:0 (*p* < 0.0001), C8:0 (*p* < 0.0001), C10:0 (*p* = 0.0012), C14:0 (*p* = 0.0002), C14:1 (*p* = 0.0002), C16:1 (*p* < 0.0001), C17:0 (*p* = 0.0006), C17:1 (*p* = 0.0256), C18:0 (*p* = 0.0003), C18index (*p* = 0.0082), C14index (*p* < 0.0001), C16index (*p* < 0.0001), C17index (*p* = 0.0022), UFA (*p* = 0.042), and total index (*p* = 0.0045, Table 2). Conversely, no significant associations were observed for C11:0, C12:0, C13:0, C15:0, C16:0, C18:1cis‐9, C20:0, SFA, and SFA/UFA (*p* > 0.05, Table 2). Notably, individuals carrying the allele C exhibited significantly higher levels of C14:1, C17:0, C18:0, and C14index compared to those with the allele T (*p* < 0.01), while showing significantly lower contents of C6:0, C8:0, C10:0, C14:0, C16:1, and C16index than the allele T (*p* < 0.05).

**TABLE 2:** Associations of the SNP g.22889812C/T (rs109915272) in *NFKB2* gene with 24 milk fatty acid traits in Chinese Holstein cows (LSM ± SE).

SNPs	Genotype (No.)	C6:0	C8:0	C10:0	C11:0	C12:0	C13:0
rs109915272	CC (385–416)	0.4866 ± 0.0126^A^	0.9481 ± 0.0110^Aa^	2.7988 ± 0.0331^A^	0.0552 ± 0.0035	2.9904 ± 0.0423	0.0977 ± 0.0030
	CT (390–420)	0.4272 ± 0.0125^B^	0.9276 ± 0.0109^Ab^	2.8059 ± 0.0326^A^	0.0568 ± 0.0035	3.0071 ± 0.0422	0.0990 ± 0.0030
	TT (89–98)	0.5463 ± 0.0178^C^	1.0039 ± 0.0149^B^	2.9363 ± 0.0443^B^	0.0628 ± 0.0044	3.0937 ± 0.0573	0.1028 ± 0.0048
	*p*	< 0.0001**	< 0.0001**	0.0012**	0.077	0.1278	0.5741

SNPs	Genotype (No.)	C14:0	C14:1	C15:0	C16:0	C16:1	C17:0
rs109915272	CC (409–418)	10.0693 ± 0.0719^Aa^	0.6932 ± 0.0189^Aa^	0.9873 ± 0.0127	34.832 ± 0.1829	1.2979 ± 0.0257^Aa^	0.572 ± 0.0033^A^
	CT (409–419)	10.21 ± 0.0712^b^	0.6505 ± 0.0184^b^	0.9981 ± 0.0125	34.8083 ± 0.1794	1.391 ± 0.0252^B^	0.5683 ± 0.0032^A^
	TT (93–98)	10.4208 ± 0.1001^B^	0.5874 ± 0.0276^B^	1.0084 ± 0.0192	34.811 ± 0.2668	1.3964 ± 0.0370^b^	0.554 ± 0.0048^B^
	*p*	0.0002**	0.0002**	0.4383	0.9869	< 0.0001**	0.0006**

SNPs	Genotype (No.)	C17:1	C18:0	C18:1cis‐9	C18index	C20:0	C14index
rs109915272	CC (350–417)	0.1877 ± 0.0026	14.2832 ± 0.0905^A^	19.0837 ± 0.1219	56.9161 ± 0.2818^A^	0.1685 ± 0.0018	6.5168 ± 0.1433^A^
	CT (346–421)	0.1926 ± 0.0025	13.9856 ± 0.0888^B^	19.2344 ± 0.1192	57.6506 ± 0.2774^B^	0.1708 ± 0.0017	6.1004 ± 0.1419^B^
	TT (75–98)	0.1858 ± 0.0037	13.8558 ± 0.1397^B^	18.9697 ± 0.1875	57.6182 ± 0.4206^AB^	0.1695 ± 0.0027	5.336 ± 0.2099^C^
	*p*	0.0256*	0.0003**	0.2204	0.0082**	0.3359	< 0.0001**

SNPs	Genotype (No.)	C16index	C17index	SFA	UFA	SFA/UFA	Total index
rs109915272	CC (411–418)	3.5484 ± 0.0661^A^	24.4824 ± 0.225^A^	68.0386 ± 0.1682	30.3462 ± 0.1526	2.2811 ± 0.0223	27.3513 ± 0.1479^a^
	CT (412–421)	3.7958 ± 0.0645^B^	25.0571 ± 0.2213^B^	67.8129 ± 0.1648	30.6235 ± 0.1501	2.2521 ± 0.0216	27.6958 ± 0.1471^b^
	TT (95–98)	3.8074 ± 0.0937^B^	25.1761 ± 0.3184^AB^	68.1674 ± 0.2529	30.1735 ± 0.2303	2.3104 ± 0.0338	27.2563 ± 0.2116^ab^
	*p*	< 0.0001**	0.0022**	0.1831	0.042*	0.1266	0.0045**

*Note:* *Indicated significant (*p* < 0.05). **Indicated highly significant (*p* < 0.01). ^a,b^Meant within a column with no common superscript letters differ at *p* < 0.05. ^A,B^Meant within a column with no common superscript letters differ at *p* < 0.01. No. was the number of animals involved in corresponding genotype.

Abbreviations: LSM = least square mean, SE = standard error.

Additionally, the additive, dominant, and allele substitution effects of the SNP g.22889812C/T on the 24 milk FAs were calculated. The analysis confirmed that g.22889812C/T exerted significant additive, dominant, or allele substitution effects on C6:0, C8:0, C10:0, C11:0, C12:0, C14:0, C14:1, C16:1, C17:0, C18:0, C20:0, C14index, C16index, C17index, UFA, SFA/UFA, and total index (Table 3; *p* < 0.05). In contrast, no significant additive, dominant, or allele substitution effects were found for C13:0, C15:0, C16:0, C18:1cis‐9, C18index, C20 index, and SFA (*p* > 0.05). These results suggested that the *NFKB2* gene had significant genetic effects on milk FAs, and the SNP g.22889812C/T might represent a causal mutation.

**TABLE 3:** Additive(a), dominant(d) and allele substitution(α) effects of the SNP g.22889812C/T (rs109915272) on milk fatty acids in Chinese Holstein cows.

SNPs	Genotype	C6:0	C8:0	C10:0	C11:0	C12:0	C13:0	C14:0	C14:1
rs109915272	a	−0.0299**	−0.0279**	−0.0688**	−0.0038*	−0.0517*	−0.0026	−0.1758**	0.0529**
	d	−0.0893**	−0.0483**	−0.0616*	−0.0021	−0.0349	−0.0013	−0.0351	0.0102
	α	0.0007	−0.0117	−0.0478**	−0.0031*	−0.0399	−0.0021	−0.1636**	0.0495**

SNPs	Genotype	C15:0	C16:0	C16:1	C17:0	C17:1	C18:0	C18:1cis‐9	C18index
rs109915272	a	−0.0106	0.0105	−0.0493**	0.009**	0.001	0.2137**	0.057	−0.3511
	d	0.0002	−0.0132	0.0439	0.0054	0.0058**	−0.0839	0.2077	0.3834
	α	−0.0106	0.015	−0.0645**	0.0072**	−0.001	0.2654**	−0.0142	−0.2204

SNPs	Genotype	C20:0	C14index	C16index	C17index	SFA	UFA	SFA/UFA	Total index
rs109915272	a	−0.0005	0.5904**	−0.1295**	−0.3469*	−0.0644	0.0864	−0.0147	0.0475
	d	0.0018	0.174	0.1179*	0.2279	−0.2901	0.3636*	−0.0437*	0.3921**
	α	−0.0012	0.5303**	−0.1704**	−0.4253**	0.0346	−0.037	0.0003	0.1811

*Note:* * and ** indicated significant effects, namely, *p* < 0.05 and *p* < 0.01, respectively.

### 4.4 Prediction of TFs Changed by the SNP g.22889812C/T

Based on our predictions, this result showed that the SNP g.22889812C/T altered the binding sites of six TFs (Table 4). Of these, the allele C of g.22889812C/T formed a transcription factor binding site (TFBS) for ZNF35 (human zinc finger protein ZNF35), whereas the T allele creates five distinct TFBSs for MYT1 (myelin transcription factor 1), NFAT5 (nuclear factor of activated T‐cells 5), E2F7 (E2F transcription factor 7), ZNF282 (zinc finger protein 282) and PRDM4 (PR domain zinc finger protein 4). Specifically, based on the ci‐value (> 60%) and whether the SNP g.22889812C/T was within the core regions of TF binding sites, ZNF282 and PRDM4 were the most promising TFs to affect the transcript activity of the *NFKB2* gene through C/T mutation on SNP g.22889812C/T.

**TABLE 4:** Changes of transcription factors (TFs) caused by the SNP g.22889812C/T (rs109915272) of NFKB2 gene.

SNP	Sequence	TF	Matrix similarity threshold	ci‐value	Core‐sequence
g.22889812C/T	TCCTCTAAGT **C** TTCCCGGCAG	ZNF35	1.00	< 60%	Yes
	TCCTCTAAGT **T** TTCCCGGCAG	MYT1	0.892	> 60%	No
		NFAT5	0.873	> 60%	No
		E2F7	0.881	< 60%	No
		ZNF282	0.831	> 60%	Yes
		PRDM4	0.718	> 60%	Yes

*Note:* The SNP in sequences was highlighted in red.

### 4.5 Luciferase Activity Caused by the SNP g.22889812C/T

To determine whether SNP g.22889812C/T affected the promoter activity of the *NFKB2* gene, a luciferase assay was implemented. The luciferase activities for both the C and T alleles were significantly elevated compared to the pGL4.14 empty vector (*p* < 0.0007, Figure 1B) and the blank control (*p* < 0.0008, Figure 1B). Of note, the luciferase activity of allele T was significantly lower than that of allele C (*p* = 0.0004; Figure 1B), suggesting that SNP g.22889812C/T markedly altered the transcript activity of the *NFKB2* gene, thereby influencing *NFKB2* expression.

### 4.6 Confirmation of TFs Changed by SNP g.22889812C/T Using EMSA and Super‐Shift EMSA

To further validate whether the SNP g.22889812C/T changed the binding of ZNF282 and PRDM4. We generated a pair of 5′‐biotinylated oligonucleotide probes containing either the wild‐type sequences or the mutated sequences. EMSA revealed that T probe corresponding to the SNP g.22889812C/T formed a detectable DNA‐protein complex (Figure 1C), while the C probe displayed a weaker complex (Figure 1C). The blank controls (CK) and a 150‐fold excess of unlabeled consensus oligonucleotide probes did not yield any detectable DNA‐protein complexes for either the C or T probes (Figure 1C). These findings indicated that SNP g.22889812C/T altered the binding affinity of TFs, with allele T exhibiting stronger DNA‐protein interactions.

Building on the prediction of TFs, the allele T established the TFs for ZNF282 and PRDM4. The super‐shift EMSA with ZNF282 and PRDM4 antibodies (Figure 1D) was conducted. The T probe of SNP g.22889812C/T revealed a DNA‐protein complex associated with ZNF282, whereas the C probe did not (Figure 1D). Neither the T nor C probes exhibited any DNA‐protein complexes associated with PRDM4 (Figure 1D). Therefore, these results suggested that SNP g.22889812C/T altered the binding interaction with TF ZNF282, thereby regulating *NFKB2* gene expression to potentially impact the content of milk FAs.

## 5 Discussion

This study served as a follow‐up research for our previous GWAS on milk FAs in Chinese Holstein [24], in which *NFKB2* was identified as a promising candidate gene for milk FAs. *NFKB2* was involved in MAPK, NF‐kappa B, and breast cancer pathways, and played a critical role in mammary gland development [29]. In the current research, the genetic effects of the *NFKB2* gene on milk FAs were confirmed in a new population of Holstein cattle, identifying its causative mutation.

This study provided novel insights into the essential role of the *NFKB2* gene in regulating milk fat metabolism in BMECs. Through siRNA‐mediated knockdown of *NFKB2*, the significant decreases in intracellular triglyceride concentration and lipid droplet secretion were observed. These results suggested that *NFKB2* directly promoted lipid synthesis in BMECs, underscoring its pivotal role in milk fat production. Our findings highlighted *NFKB2* as a critical regulatory factor in mammary lipid biosynthesis. Knockdown of *NFKB2* led to a significant downregulation of 18 key lipid metabolism‐related genes. Notably, these genes are part of multiple key signaling pathways, including PI3K‐AKT, NF‐κB, MAPK, adipocytokine, PPAR, and AMPK signaling pathways, all of which play central roles in lipid uptake, synthesis, and storage [30, 31, 32, 33, 34, 35]. This study highlighted *NFKB2* as a key regulator of lipid synthesis and secretion in BMECs, acting through its influence on critical genes and pathways associated with fatty acid metabolism. These findings not only advanced our understanding of the molecular regulation of milk fat production but also provided a foundation for future studies aimed at genetic or nutritional interventions to improve the composition and nutritional value of milk fat. Further exploration of *NFKB2*'s role in mediating the cross‐regulation between inflammation and metabolism could uncover additional insights into its potential as a target for optimizing milk quality and production efficiency.

The identification of causal mutations is crucial in various fields such as genetics, medicine, and evolutionary biology [36]. The identification of the SNP g.22889812C/T in the upstream region of the *NFKB2* gene represented a significant advancement in our understanding of the genetic factors influencing fatty acid traits. When the mutant genotype was CC, there were marked increases in the contents of C14:1, C17:0, C18:0 and C14index FAs, while the TT genotype was associated with the significant elevation in the content of C6:0, C8:0, C10:0, C14:0, C16:1, and C16index. These indicated a clear relationship between this specific mutation and metabolic pathways related to lipid biosynthesis. Moreover, the distinct fatty acid variations associated with the genotypes underscore the potential for utilizing genetic markers in the selective breeding of animals to enhance desirable traits related to fat composition, which is relevant in both human health and industry. SFAs intake is consistently linked to an elevated risk of cardiovascular diseases in epidemiological and clinical studies [8, 9]. Experimental studies demonstrate that dietary SFAs, induce astrocytic apoptosis and pro‐inflammatory cytokine release, ultimately disrupting dopaminergic signaling and elevating depression susceptibility [37]. Notably, human epidemiological studies present conflicting findings regarding this association. A longitudinal investigation identified significant correlations between elevated SFA intake and depressive symptomatology in perimenopausal women [38], whereas a population‐based cross‐sectional analysis found no statistically robust association following energy intake normalization [39]. The heterogeneity in reported outcomes might be attributable to differential chain‐length compositions of dietary saturated FAs. Recent data showed possible beneficial effects of short‐chain SFAs and medium‐chain SFAs on health [40]. Increased consumption of short‐chain SFAs demonstrated a protective effect against dyslipidemia and diabetes in the study population [40]. This genetic marker of SNP g.22889812C/T held significant potential for application in selective breeding programs aimed at optimizing fat composition in animals, with implications for both human health and industrial applications. Future research should focus on elucidating the mechanistic pathways underlying these associations and exploring the potential for targeted dietary interventions and genetic selection to mitigate adverse health effects while maximizing beneficial outcomes.

Transcriptional activation is precisely orchestrated through multifaceted regulatory mechanisms involving allosteric modulation of transcription factor‐coactivator assemblies, and combinatorial post‐translational modifications [41]. As sequence‐specific DNA‐binding proteins, TFs regulate gene expression by interacting with promoter elements [42], thereby governing critical cellular events spanning differentiation pathways [43], proliferation cycles [44], and apoptotic mechanisms [45]. ZNF282, a zinc finger‐containing transcriptional regulator, was initially discovered through its specific interaction with the HTLV‐I U5RE, a cis‐acting element critical for viral replication control [46]. ZNF282 functions as a co‐activator of estrogen receptors and is crucial in the process of breast tumorigenesis [41, 47]. In this study, the computational predictions, luciferase assays, EMSA and super‐shift EMSA revealed that the allele T of SNP g.22889812C/T bonded with ZNF282, which inhibited the *NFKB2* expression, ultimately, enhanced C6:0, C8:0, C10:0, C14:0, C16:1, and C16index accumulation and reduced C14:1, C17:0, C18:0, and C14index levels. Future studies should explore the broader network of pathways regulated by *NFKB2* and ZNF282, as well as investigate potential environmental interactions that could modulate the effects of this SNP on lipid metabolism.

## 6 Conclusion

In conclusion, this study, employing a post‐GWAS approach, first demonstrated significant genetic associations between the *NFKB2* gene and milk FAs in dairy cattle. Furthermore, the research identified the allele T of SNP g.22889812C/T changed the binding of TF ZNF282 to decrease the promoter activity of the *NFKB2* for reducing milk fat content. This study provided a key molecular marker for dairy cattle breeding.

## Author Contributions

D.S.: conceived and designed the experiments. L.S.: performed the experiments and analyzed the data. Z.G., N.S., C.L., and B.H.: contributed the materials. D.S. and L.S.: wrote the paper.

## Ethics Statement

All protocols were reviewed and approved by the Institutional Animal Care and Use Committee (IACUC) at China Agricultural University (Permit Number DK996). Milk, blood, and semen samples were collected specifically for this study following standard procedures with the full agreement of the Beijing Sanyuanlvhe Dairy Farming Center who owned the Holstein cows and bulls.

## Conflicts of Interest

The authors declare no conflicts of interest.

## Supporting information


**Table S1:** Quantitative real‐time PCR (qRT‐PCR) primer information for *NFKB2*, *GAPDH*, *MAVADL1* and 18 lipid related genes.
**Table S2:** PCR primer information of *NFKB2* gene.

## Data Availability

The data that support the findings of this study are available in the methods and/or Supporting Information of this article.
